# Polysaccharides From the Roots of *Millettia Speciosa* Champ Modulate Gut Health and Ameliorate Cyclophosphamide-Induced Intestinal Injury and Immunosuppression

**DOI:** 10.3389/fimmu.2021.766296

**Published:** 2021-10-21

**Authors:** Xiaogang Chen, Wenjing Sun, Baichang Xu, Enyun Wu, Yao Cui, Kaiyuan Hao, Geyin Zhang, Congcong Zhou, Yanping Xu, Jiang Li, Hongbin Si

**Affiliations:** College of Animal Sciences and Technology, Guangxi University, Nanning, China

**Keywords:** *Millettia Speciosa* Champ, polysaccharides, cyclophosphamide, intestinal barrier injury, immunoregulation, gut microbiota

## Abstract

Cyclophosphamide (CTX), a common anticancer drug, can cause a variety of side effects such as immunosuppression and intestinal mucosal injury. Polysaccharides are the major bioactive components of the roots of *Millettia Speciosa* Champ and have gained attention for their immunomodulatory activity. This study was designed to evaluate the immunomodulatory effect of *Millettia Speciosa* Champ polysaccharide (MSCP) on CTX-induced mice and the possible mechanism. The results showed that MSCP attenuated the CTX-induced decrease in body weight and immune organ indices in mice and promoted the secretion of immune-related cytokines (IL-2, IL-4, IL-10, TNF-α, and IgG). Meanwhile, MSCP restored intestinal morphology, increased the ratio of villus height/crypt depth (V/C), and improved the number of goblet cells and mucins expression. At the mRNA level, MSCP activated the TLRs/MyD88/NF-κB p65 pathway and enhanced the expression of genes related to intestinal mucosal integrity (Occludin1, Claudin1, and MUC-2). In addition, MSCP as a prebiotic improved microbial community diversity, regulated the relative abundance of dominant microbiota from the phylum level to the genus level, restored CTX-induced gut microbial dysbiosis, and promoted short-chain fatty acid production in mice. Based on the present findings, MSCP may modulate the immune response depending on enhancing intestinal health, suggesting that MSCP holds promise as a promising immunostimulant in functional foods and drugs.

## Introduction

The prevalence of cancer is rising worldwide, and chemotherapy is widely used as a treatment for tumor growth (1). Cyclophosphamide (CTX) is a chemotherapeutic agent commonly used to treat malignant tumors and autoimmune diseases (2). However, like other chemotherapy drugs, CTX has some problems that cannot be ignored, including causing immunosuppression and various toxic side effects. Long-term use of CTX causes decreased immune function and damage to the gastrointestinal mucosal barrier, leading to an increased risk of immunodeficiency and secondary infections (3–6). In addition, studies have shown that CTX can alter the intestinal microbiota, resulting in a significant enrichment of pathogenic microorganisms such as *Escherichia coli* and *Enterobacteriaceae* in feces (7, 8). Therefore, it is urgent to develop a safe and effective immunomodulator to reduce the side effects and improve the efficacy of CTX.

The immune system is a powerful defense mechanism protecting the body from pathogens and infections, maintaining immune homeostatic balance under normal physiological conditions. The intestine is the body’s largest digestive and absorption organ and a vital immune organ (9). The intestine possesses a defense barrier consisting of intestinal epithelial cells (IECS) tightly connected to prevent the invasion of pathogenic microorganisms. The intestinal barrier contains a variety of immune cells, including T cells, B cells, innate lymphoid cells (ILCs), and monocyte-macrophage systems (monocytes, DC cells, and macrophages) (10–12). In addition to this, the intestine contains a variety of antigens (from pathogenic bacteria, commensal bacteria, and foods), resulting in a gut immune system with some regulatory mechanisms that differ from the systemic immune system and play an essential role in maintaining intestinal health (13, 14).

The intestinal microbiota is closely related to the intestinal immune system, and their interactions, which influence the development of disease, have attracted widespread attention (15). Approximately 100 trillion microorganisms live in the microbiota of the human gut, which is what determines the mutually beneficial relationship between the gut and microbiota (16). Specifically, the gut provides nutrition and a living environment for the microbiota, which plays a role in intestinal metabolism, nutrition, and immunity (17). Once this mutually beneficial relationship is disrupted, disruption of the intestinal immune system and dysbiosis of the microbiota can lead to a diverse range of human diseases, such as inflammatory bowel disease (18). Therefore, maintaining a healthy gut is a crucial task.

In recent years, developing natural polysaccharides to protect intestinal health has become a topic of intensive research. Polysaccharides have physiological functions such as immune enhancement, antioxidant, antitumor, anti-inflammatory, and maintenance of intestinal health (19). Food-derived natural polysaccharides have been shown to modulate immune responses by acting directly on immune cells, improving microbiota, and promoting the production of short-chain fatty acids (11). Examples include polysaccharides extracted from *Dendrobium huoshanense*, *Cordyceps sinensis*, and *Lycium barbarum*, which can stimulate cytokine production, improve the intestinal mucosal barrier and promote the proliferation of beneficial microorganisms that produce short-chain fatty acids (20–22).

*Millettia speciosa* Champ. (*M. speciosa*) is a plant of the *Millettia* genus belonging to the Leguminosae family, is a well-known medicinal herb for both food and medicine in the Lingnan region of China (23). It has a long history of medicinal use and is commonly used to treat chronic bronchitis, hepatitis, rheumatoid arthritis, and diabetes (24). Recent studies have shown that *M. speciosa* has immunomodulatory, anti-fatigue, antioxidant, hypoglycemic, and hypolipidemic effects (24–26). Polysaccharide is the main bioactive component of *M. speciosa* roots (MSCP), and *in vitro* immunomodulatory assays have demonstrated that MSCP2 could improve phagocytosis and stimulate cytokine and NO production (26). However, we found that few studies on the *in vivo* immune activity of MSCP have been reported. Therefore, this study aimed to evaluate the protective effects of MSCP against CTX-induced immunosuppression and intestinal injury in mice. In addition, 16S-rDNA sequencing was employed to explore the regulatory effects of MSCP on the intestinal microbiota. The results of this study will reveal the immunomodulatory and intestinal epithelial protective effects of MSCP, and the intestinal health of the organism is an essential factor in its role.

## Materials and Methods

### Materials and Reagents

*M. speciosa* was from the local market (Nanning, China). Cyclophosphamide was purchased from Sigma-Aldrich (St. Louis, MO, USA. Purity > 99%). *Astragalus* polysaccharide purchased from Guilin Huayi Health Science and Technology Co., Ltd. (Guilin, China, purity ≥65%). HE and AB-PAS dye solution set were obtained from Servicebio technology Co., Ltd. (Wuhan, China). Mouse IL-2, IL-4, IL-10, TNF-α, IgG ELISA kits were purchased from Jiangsu Meimian industrial Co., Ltd. (Yancheng, Jiangsu, China). TRIzol reagent, First-strand cDNA Synthesis Mix and RealStar Green Fast Mixture were purchased from GenStar Co., Ltd. (Beijing, China). All SCFA standards, excluding propionate (Wokai^®^, Shanghai, China. Purity 99.5%), were purchased from Sigma-Aldrich (Shanghai, China. Purity > 99%). A series of standard monosaccharides (fucose, rhamnose, arabinose, galactose, glucose, xylose and mannose, etc.) were purchased from the Sigma (St. Louis, MO, USA). All other reagents in the experiments were purchased from China and were analytically pure.

### Preparation of Polysaccharide (MSCP)

*M. speciosa* root pieces (1000 g) were weighed, dried, crushed into powder. Deionized water (1: 10, w/v) was added and extracted three times at 95-100°C for 1.5 h. After filtration, the extracts were combined and concentrated to 1000 ml under reduced pressure, anhydrous ethanol was added and left to stand at 4°C for 24 h. The precipitate obtained by centrifugation was dissolved in pure water and the proteins were removed using Sevag reagent (chloroform: n-butanol = 4:1, v/v). The precipitation process was repeated with anhydrous ethanol and acetone. Finally, the precipitate was freeze-dried to obtain MSCP.

### Physicochemical Features Analysis of MSCP

The total carbohydrate content of MSCP was measured by phenol-sulfuric acid method (27). The Mw of MSCP were determined by high-performance gel permeation chromatography (HPGPC) on a Shimadzu LC-10A system equipped with a BRT105-104-102 column (8 × 300 mm, Borui Saccharide, Biotech. Co. Ltd.) and a parallax detector. Calibration curves for the calculation of molecular weight were plotted according to the method of Wang et al. (28). The monosaccharide composition analysis was processed according to the method described by Wang et al (28). The monosaccharide composition of MSCP was analyzed using ICS5000 ion chromatogram equipped with an electrochemical detector (Thermo Fisher, USA). The assay was analyzed with reference to standard monosaccharides including fucose, rhamnose, arabinose, galactose, glucose, xylose, mannose, fructose, ribose, galacturonic acid and glucuronic acid. The organic functional groups present in MSCP were determined by Fourier transform infrared spectroscopy (FT-IR). The dried polysaccharides and KBr powder were mixed and the mixture was pressed into sheets for recording with a Fourier transform infrared spectrometer FT-IR650 in the range of 4000-400 cm^-1^.

### Animals and Experimental Design

Kunming mice (half male and female,20 ± 2g) were purchased from Changsha Tianqin Biotechnology Co., Ltd. (Changsha, China, Certificate number: SCXK(Xiang)2019-0014). All mice were acclimatized for one week before the experiment (temperature 22 ± 2°C, relative humidity 50 ± 5%, and 12 h light/dark cycle) and fed standard chow and water ad libitum. All animal experimental protocols for this experiment were approved by the Institutional Animal Care and Use Committee of Guangxi University (Nanning, China) [No. Gxu-2021-129] and were performed in accordance with the Guide for the Protection and Use of Laboratory Animals of the National Institutes of Health.

After acclimation, mice were randomly divided into 6 groups (n=12): normal control group (NC), CTX model group (MC), low dose group (MSCP-L), middle dose group (MSCP-M), high dose group (MSCP-H) and *Astragalus* polysaccharides group (AMP)(**Table 1**). For the next 14 days, mice in the NC and MC groups were administered saline daily, while the low, medium and high dose groups were administered 100, 200 and 400 mg/kg/d MSCP, respectively. Mice in the AMP group were given 400 mg/kg/d AMP. At days 10, 12 and 14, mice in all groups except the NC group were injected intraperitoneally with 80 mg/kg.bw cyclophosphamide, and the NC group was injected with an equal volume of saline. After the last administration, mice were fasted with food but not water for 12 hours and weighed. All mice were anesthetized with CO_2_ and euthanized with cervical dislocation. Blood, spleen, thymus, small intestine and cecum contents were collected for further analysis.

**Table 1:** Different treatment groups in mice.

Group	Treatment	Duration
NC	Normal saline (gavage)	Days 1–14
MC	80 mg/kg.bw CTX (inject)	Days 10, 12 and 14
MSCP-L	100 mg/kg/d MSCP (gavage)	Days 1–14
	80 mg/kg.bw CTX (inject)	Days 10, 12 and 14
MSCP-M	200 mg/kg/d MSCP (gavage)	Days 1–14
	80 mg/kg.bw CTX (inject)	Days 10, 12 and 14
MSCP-H	400 mg/kg/d MSCP (gavage)	Days 1–14
	80 mg/kg.bw CTX (inject)	Days 10, 12 and 14
AMP	400 mg/kg/d AMP (gavage)	Days 1–14
	80 mg/kg.bw CTX (inject)	Days 10, 12 and 14

NC, normal control; MC, cyclophosphamide; MSCP-L, pretreatment-low dose group with 100 mg/kg/d MSCP; MSCP-M, pretreatment-middle dose group with 200 mg/kg/d MSCP; MSCP-H, pretreatment-high dose group with 400 mg/kg/d MSCP; AMP, pretreatment with 400 mg/kg/d AMP.

### Determination of Body Weight and Organ Indices

The mice in each group were weighted and executed at the end of the experiment. The spleen and thymus were removed and weighed. The organ index was calculated according to the following formula: Organ index (mg/g) = weight of spleen or thymus (mg)/mouse body weight (g).

### Cytokines Detection by ELISA

Blood samples were collected and serum was isolated, and IL-2, IL-4, IL-10, TNF-α, and IgG levels were measured using ELISA kits. 100 mg of small intestine tissue was mixed with 900 uL of phosphate buffered saline (PBS) and homogenized thoroughly, centrifuged for 20 min (3000 rpm, 4°C), and the supernatant was collected to detect IL-2, IL-4, IL-10 and TNF-α levels. The detection method was performed according to the instructions provided by the ELISA manufacturer.

### Histological Examination

The spleen and jejunum tissue were fixed with 10% neutral formalin for 24 hours, dehydrated with alcohol, and then processed into paraffin-embedded blocks. Sections (3-5 μm thick) were stained with hematoxylin and eosin (H&E) to visualize histological changes. Images were acquired with an Eclipse Ci-L photomicroscope (Nikon, Japan) (100× magnification). The height of five intact villi and the depth of five crypt foci in each jejunum tissue section were measured using Image-Pro Plus 6.0 analysis software, and the mean values were calculated. The ratio of villi length to crypt depth was calculated as an index to assess small intestine injury.

The number of goblet cells and mucin area of jejunum tissue were measured using the alcain blue-periodic acid schiff (AB-PAS) staining method. The jejunum tissues were stained and then imaged at 100x using a photomicroscope, and the number of goblet cells and mucin area were measured with Image-Pro Plus 6.0 software. Five intact villus epithelia were selected from each image, their lengths were measured, and the numbers of goblet cells on the corresponding epithelia were counted. Unit length goblet cell number = goblet cell number/villus epithelium length.

### RNA Extraction and qRT-PCR Analysis

Total RNA of small intestine tissue was extracted by TRIzol reagent, and cDNA was synthesized using the StarScript II First-strand cDNA Synthesis Mix (With gDNA Remover) according to the manufacturer’s instructions. The PCR reactions were performed using RealStar Green Fast Mixture on the LightCycler 96 System (Roche). The β-actin was used as a housekeeping gene to normalize the gene expression. Relative expression levels were analyzed by 2^-ΔΔCt^ calculation method. The primer sequences (Sangon Biotech Co., Ltd, China) used in this study were shown in **Supplementary Table S1**.

### Genomic DNA Extraction and 16S-rDNA Sequencing of Feces

The genomic DNA of cecal contents was extracted by an E.Z.N.A.^®^ soil DNA Kit (Omega Bio-Tek, Norcross, GA, U.S.) following the manufacturer’s instruction and detected by 1% agarose gel electrophoresis. The V3-V4 hypervariable regions of the bacterial 16S-rDNA gene were amplified by universal primers 338F (5′- ACTCCTACGGGAGGCAGCAG-3′) and 806R (5′-GGACTACHVGGGTWTCTAAT-3′). PCR was performed in a 20 μL mixture, and all products were purified by AxyPrep DNA Gel Recovery kit (Axygen, Union City, USA) and quantified by QuantiFluor™ -ST (Promega, USA). PE library was build up using TruSeqTM DNA Sample Prep Kit and sequenced by an Illumina Miseq PE300 platform (Illumina, SD, USA) according to the standard scheme of Majorbio Bio-Pharm Technology Co., Ltd. (Shanghai, China). High quality sequence clustering was performed using UPARSE software (v7.0.1090, http://drive5.com/uparse/) to obtain Operational Taxonomic Units (OTUs) based on 97% similarity. Alpha-diversity and beta-diversity were analyzed according to the abundances of OTUs using the R package. The relative abundance of the dominant bacteria at phylum and genus levels were analyzed. Genus level classifications of bacteria were compared with the Wilcoxon rank sum test. The linear discriminant analysis (LDA) and LDA effect size (LEfSe) were used to analyze the dominance of bacterial communities among the groups (http://huttenhower.sph.harvard.edu/galaxy/root?tool_id=lefse_upload).

### Determination of Contents of SCFAs

Cecal contents (25 mg) were complemented with 500 μL of water [contained 0.5% phosphoric acid and 50 μg/mL of 2-ethylbutyric acid (internal standards)], and then extracted according to the manufacturer’s protocol (Majorbio Bio-Pharm Technology Co., Ltd., Shanghai, China). The contents of SCFAs were determined using an Agilent 8890B-5977B GC–MS system (Agilent Technologies Inc. CA, UAS) equipped with a HP FFAP column (30m×0.25mm×0.25μm, Agilent J&W Scientific, Folsom, CA, USA). The ion fragments of target SCFAs were automatically identified by MassHunter quantitative software (Agilent Technologies Inc., USA, No. V10.0.707.0), and the SCFAs content was calculated by standard curve. SCFAs standards were mixtures of acetic acid, propanoic acid, butanoic acid, isobutyric acid, valeric acid, isovaleric acid, hexanoic acid and isohexanoic acid.

### Statistical Analysis

All of the experimental data in the figures and tables were presented as mean ± SD, and a minimum of three independent experiments were performed. Differences among various groups were evaluated by One-way ANOVA followed by the LSD test. SPSS statistical software 22(SPSS Inc., Chicago, IL, USA) was used for statistical analysis. The value of *p* < 0.05 were considered as statistically significant.

## Results

### Physicochemical Features of MSCP

**Figure 1:**
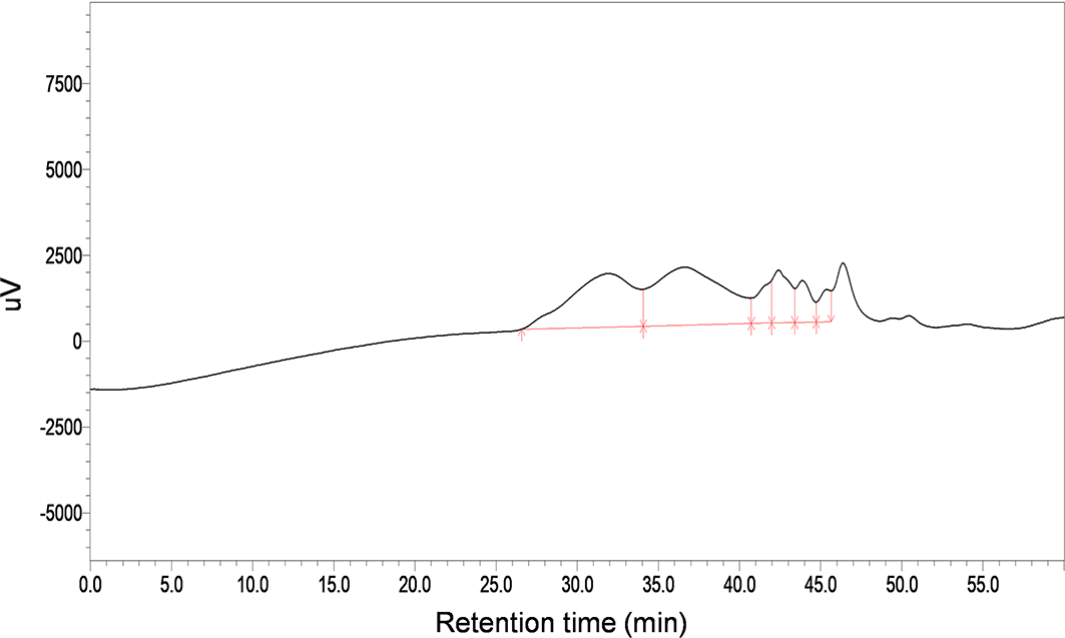
HPGPC profiles of MSCP.

**Figure 2:**
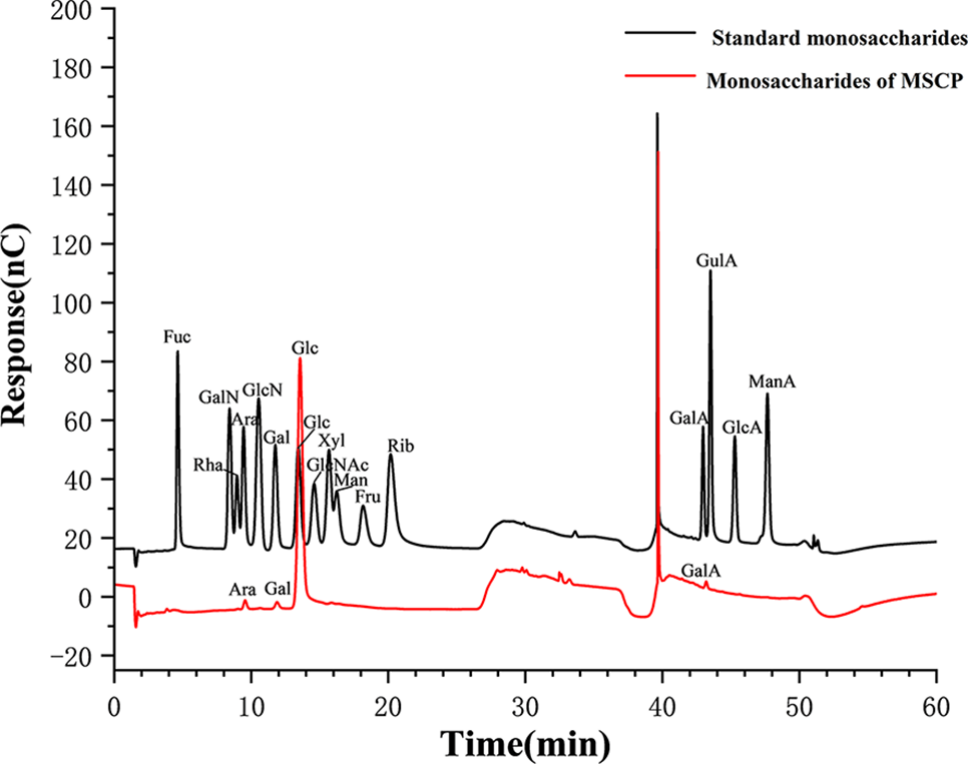
Ion chromatogram data of monosaccharide composition.

**Figure 3:**
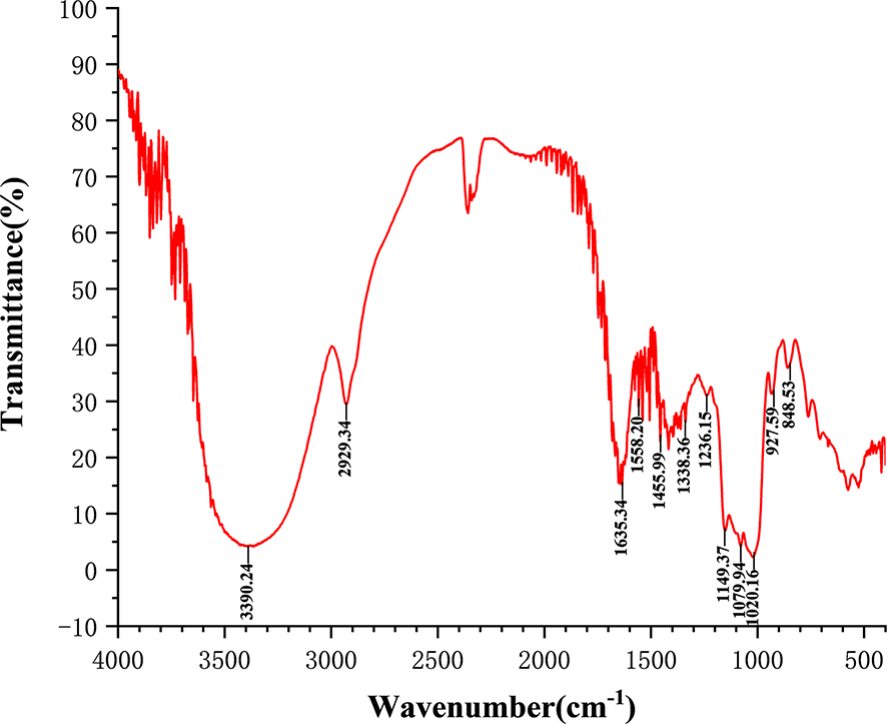
Fourier-transform infrared (FT-IR) spectra of MSCP in the range of 4000–400 cm^-1^.

The total carbohydrate content of MSCP was 78.2%, as measured by phenol sulfuric acid. The molecular weight (Mw) of MSCP was determined using the average molecular weight of dextran as the standard. **Figure 1** shown six fractions in MSCP, and their Mw were calculated as 932.325 kDa, 121.763 kDa, 11.944 kDa, 9.929 kDa, 5.268 kDa, and 2.745 kDa, respectively. This indicated a wider variety of polysaccharides in MSCP. The monosaccharide composition of MSCP was determined by Ion chromatography. As shown in **Figure 2**, MSCP was a heteropolysaccharide and mainly consisted of arabinose, galactose, glucose and galacturonic acid at a molar ratio of 2.2: 2.1: 93.8: 1.9, which indicated that glucose was the main monosaccharide type. The FT-IR spectrum of MSCP is shown in **Figure 3**. The absorption peaks observed around 3390 cm^-1^ in the spectrum of MSCP could be considered to be caused by O-H stretching vibration. The characteristic absorption at 2929 cm^-1^ and 1455 cm^-1^ was due to C-H stretching and bending vibrations. The band at 1635 cm^-1^ could be attributed to bound water (28). The peaks at 1558 cm^-1^ and 1338 cm^-1^ indicated the presence of the asymmetrical and symmetrical C=O stretching vibration. An absorption band in the region of 1400-1000 cm^-1^ was ascribed to the C-O-C stretching vibration. Peaks at 927 cm^-1^ indicated the presence of a pyranoid ring (14). Peaks at 848 cm^-1^ in the spectrum of MSCP indicated the α-configuration of sugar units (29). These results suggest that MSCP has the characteristic adsorption of typical polysaccharides.

### Effect of MSCP on Body Weight and Immune Organ Indices

The spleen and thymus are critical immune organs, and the immune organ index is known as a primitive indicator of immune competence (30). The mouse body weight, spleen, and thymus indices are listed as shown in **Table 2**. Body weight, spleen, and thymus indices in the MC group were significantly lower than in the NC group (*p* < 0.001). The spleen indices of mice in all dose groups of MSCP were significantly higher than that in MC group (*p* < 0.05), while the thymus indices of mice in MSCP-H and MSCP-M groups were significantly higher than that in MC group (*p* < 0.05). In addition, AMP at a dose of 400 mg/kg/d significantly increased both indices in mice compared with MC group (*p* < 0.05). This indicates that MSCP can effectively improve cyclophosphamide-induced weight loss and prevent atrophy of the body’s immune organs.

**Table 2:** Effects of MSCP on body weight and immune organ indices in immunosuppressive mice.

Group	Initial weight (g)	Final weight (g)	Thymus index (mg/g)	Spleen index (mg/g)
NC	22.40 ± 1.03	32.14 ± 2.53	3.18 ± 0.27	3.69 ± 0.47
MC	22.62 ± 1.16	28.58 ± 2.15^###^	0.83 ± 0.19^##^	1.20 ± 0.12^###^
MSCP-L	22.93 ± 1.27	31.20 ± 2.33^**^	1.08 ± 0.21	1.55 ± 0.17^**^
MSCP-M	22.60 ± 1.20	30.94 ± 2.43^*^	1.11 ± 0.22^*^	1.84 ± 0.32^***^
MSCP-H	22.36 ± 1.24	31.36 ± 2.72^**^	1.21 ± 0.17^**^	1.60 ± 0.24^*^
AMP	22.51 ± 1.32	30.82 ± 1.82^*^	1.17 ± 0.24^*^	1.64 ± 0.26^**^

Data are expressed as mean ± SD. ^##^p < 0.01, ^###^p < 0.001 compared with NC group; ^*^ p< 0.05, ^**^p < 0.01, ^***^p < 0.001 compared with MC group.

### Effects of MSCP on Levels of IL-2, IL-4, IL-10, TNF-α and IgG

**Figure 4:**
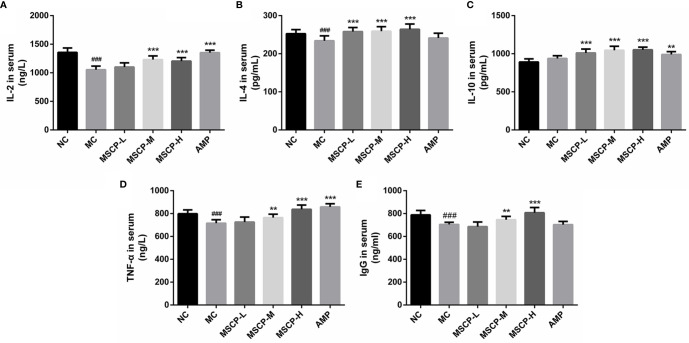
Effects of MSCP on the levels of IL-2 **(A)**, IL-4 **(B)**, IL-10 **(C)**, TNF-α **(D)** and IgG **(E)** in serum of mice. Data are expressed as mean ± SD. ^###^*p* < 0.001 compared with NC group; ^**^p < 0.01, ^***^p < 0.001 compared with MC group.

The immunomodulatory effects of MSCP on mice were observed by measuring the levels of cytokines and immunoglobulins in serum. As shown in **Figure 4**, IL-2, IL-4, TNF-α, and IgG levels in the serum of MC group were significantly lower than those of the NC group (*p* < 0.01, *p* < 0.001), suggesting that CTX has the inhibitory effect on the immune activity. This phenomenon was significantly reversed in both MSCP-M and MSCP-H groups compared with MC group (*p* < 0.01 and *p* < 0.001). The serum levels of IL-4 and IL-10 were significantly higher in the MSCP-L group than in the MC group (*p* < 0.001), and IL-2 and TNF-α secretion levels were elevated, but there was no significant difference. In addition, serum IL-2, IL-10, and TNF-α secretion were significantly higher in the AMP group compared with the MC group (*p* < 0.001). The results suggest that MSCP and AMP can improve CTX-induced immunosuppression and enhance immunity by promoting the production of cytokines and IgG.

**Figure 5:**
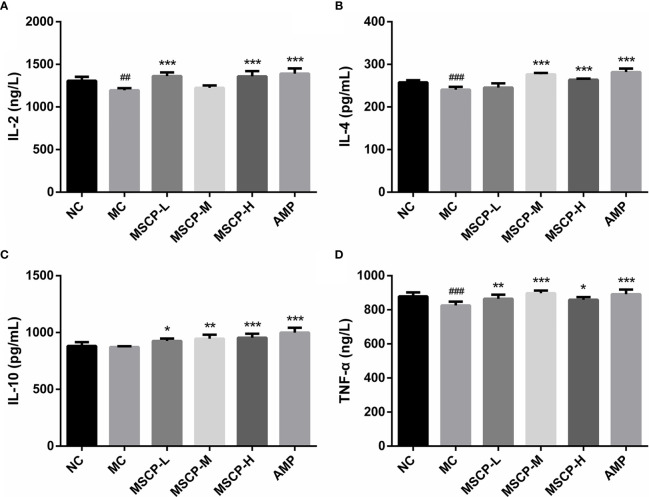
Effects of MSCP on the levels of cytokines in intestine of mice. **(A)** IL-2; **(B)** IL-4; **(C)** IL-10; and **(D)** TNF-α. Data are expressed as mean ± SD. ^##^*p* < 0.01, ^###^*p* < 0.001 compared with NC group; ^*^p < 0.05, ^**^p < 0.01, ^***^p < 0.001 compared with MC group.

To assess the effects of MSCP on intestinal mucosal immunity, the secretion of IL-2, IL-4, IL-10, TNF-α in the small intestine was detected (**Figure 5**). The MC group showed significantly lower levels of IL-2, IL-4, and TNF-α in the small intestinal tissues than the NC group (p < 0.01). IL-10 secretion was lower in the MC group than in the NC group, but that difference was insignificant. However, continuous administration of MSCP and AMP could obviously promote the secretion of the above cytokines in the small intestine, and especially the MSCP-H group showed a significant effect on the release of IL-2, IL-4, IL-10, TNF-α in small intestinal tissues (p < 0.05, p < 0.001). The results showed that, to some extent, dietary supplementation of high dose MSCP (400 mg/kg/d) significantly increases the intestinal secretion of IL-2, IL-4, IL-10, and TNF-α, which protects intestinal mucosa.

### MSCP Ameliorated Pathological Injury in the Spleen and Intestine

**Figure 6:**
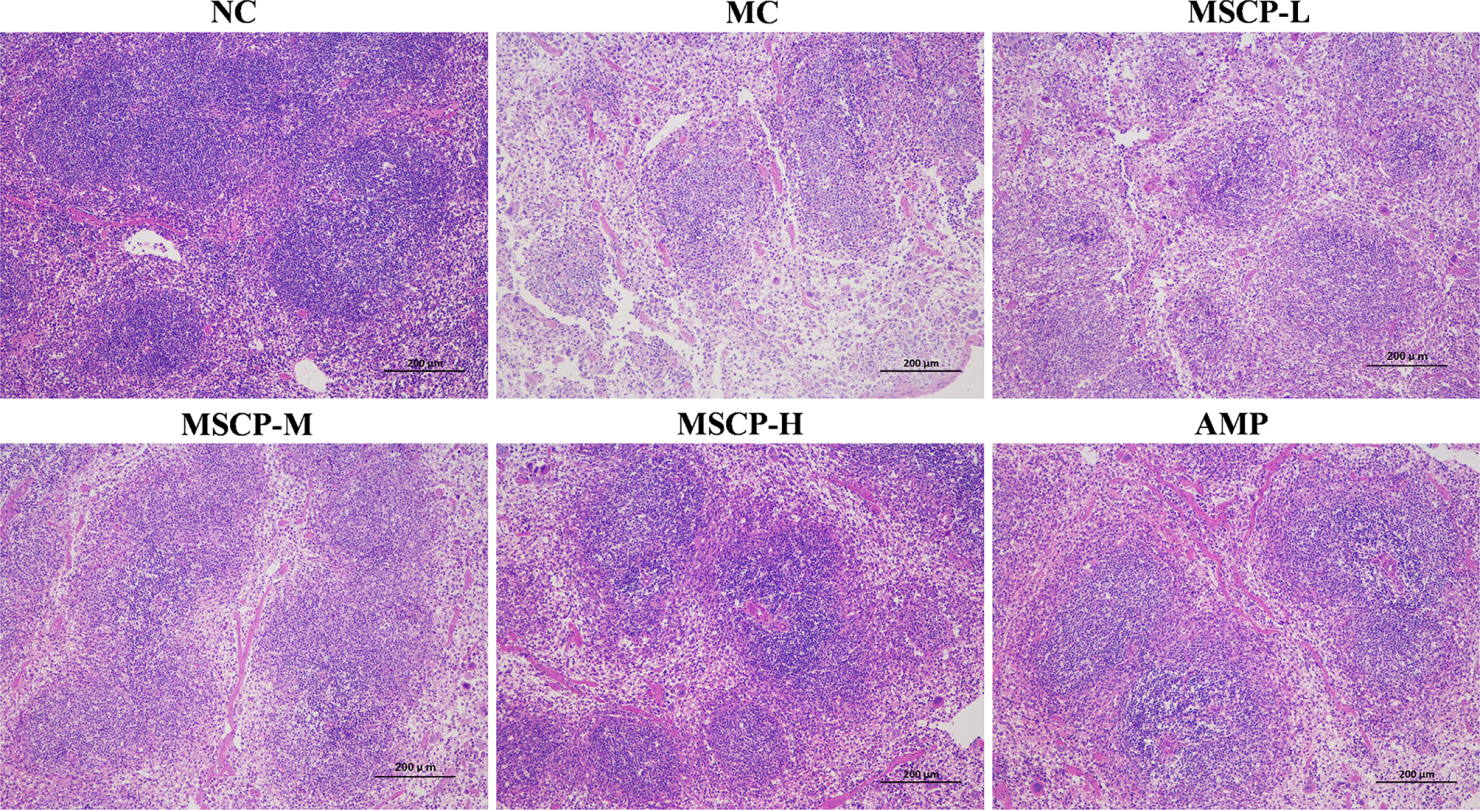
Effects of MSCP on the spleen tissues shown in HE-stained histopathological images. (100 ×, Scale bar = 200 mm).

The spleen is an important immune organ in the body, and disruption of the immune system is often accompanied by damage to the immune organs. In the present study, the histopathological images of H&E staining are shown in **Figure 6**. The splenocytes in the NC group were dense and arranged in an orderly manner, with clear nuclei and clear borders of the red and white pulp. The splenocytes in the MC group were sparse, disorganized, with nuclear consolidation and borders of the red and white pulp were inconspicuous. After MSCP and AMP intervention, the damage was gradually recovered. Splenocytes in the MSCP-H and AMP groups were dense, neatly arranged, with clear nuclei and obvious demarcation lines, similar to those in the NC group. The results showed that MSCP could effectively alleviate cyclophosphamide-induced spleen injury in mice in a dose-dependent manner.

**Figure 7:**
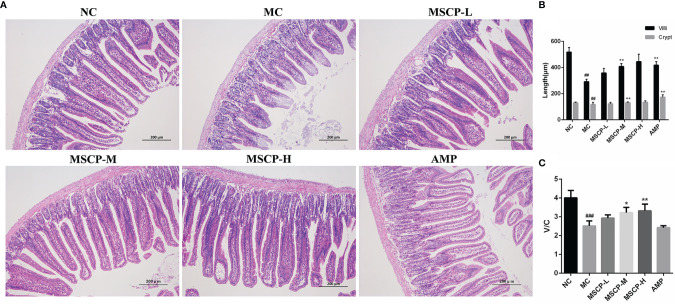
Effects of MSCP administration on the intestinal mucosal morphology in CTX-treated mice. **(A)** Hematoxylin-eosin staining of jejunum section (100 ×, Scale bar = 200 mm). **(B)** The villus height and crypt depth. **(C)** The ratio of villus height to crypt depth. Data are expressed as mean ± SD. ^##^*p* < 0.01, ^###^*p* < 0.001 compared with NC group; ^*^p < 0.05, ^**^p < 0.01, compared with MC group.

Many studies have reported that intestinal mucosal injury induced by cyclophosphamide treatment can lead to apoptosis of intestinal crypt cells, resulting in a decrease in villi height and crypts depth (31, 32). To investigate the effect of MSCP on intestinal pathological injury after CTX-treated mice, we performed an H&E analysis (**Figure 7**). The histological morphology of jejunum in NC group mice was normal, and the villi were thin, intact and neatly arranged. In contrast, the intestinal mucosa of MC group was severely damaged, showed obvious cell necrosis and edema, villus atrophy, shallower crypt and looser structure. These phenomena were alleviated in MSCP-treated mice, and the villi were elongated and tightly arranged (*p* < 0.01). In addition, the villi height/crypt depth (V/C) ratio was significantly higher in both the MSCP-M and MSCP-H groups compared with the MC group (*p* < 0.05, *p* < 0.01).

**Figure 8:**
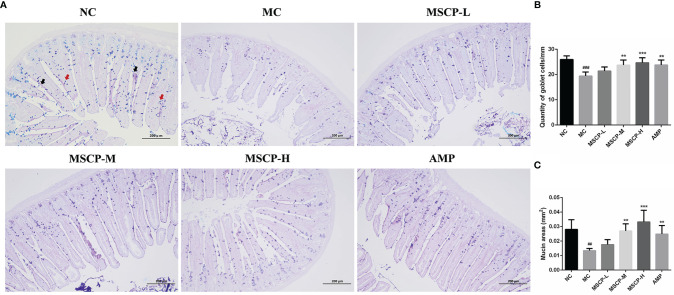
The goblet cells and mucins area were observed by AB-PAS staining in the jejunum. **(A)** AB-PAS staining of jejunum section (100 ×, Scale bar = 200 mm). Red arrow represents the goblet cells and black arrow represents the mucins. **(B)** The quantity of goblet cells/mm. **(C)** Mucin areas (mm^2^). Data are expressed as mean ± SD. ^##^*p* < 0.01, ^###^*p* < 0.001 compared with NC group; ^**^p < 0.01, ^***^p < 0.001 compared with MC group.

The jejunum sections stained with AB-PAS, as shown in **Figure 8**, the number of goblet cells and mucin area were significantly decreased in MC group compared with NC group (*p* < 0.001, *p* < 0.01). However, the above two indices were significantly increased in mice given medium and high doses of MSCP and AMP (*p* < 0.01, *p* < 0.001), especially in MSCP-H group (*p* < 0.001). The above results indicated that MSCP could reduce cyclophosphamide-induced intestinal tissue damage in a dose-dependent manner.

### Effects of MSCP on Relative Gene Expression

**Figure 9:**
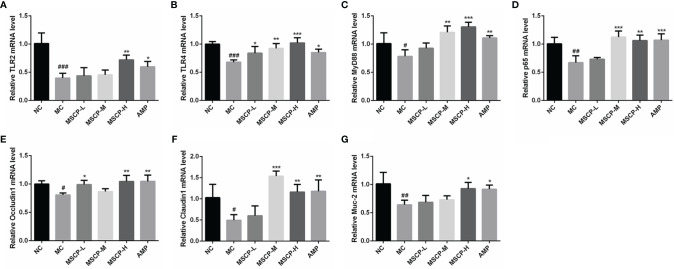
Effects of the treatments of MSCP (100, 200 and 400 mg/kg· BW/d, respectively) and AMP (400 mg/kg· BW/d) on the relative mRNA expression of **(A)** TLR2, **(B)** TLR4, **(C)** MyD88, **(D)** p65, **(E)** Occludin1, **(F)** Claudin1 and **(G)** MUC-2 in small intestine tissues. Data are expressed as mean ± SD. ^#^*p* < 0.05, ^##^*p* < 0.01, ^###^*p* < 0.001 compared with NC group; ^*^p < 0.05, ^**^p < 0.01, ^***^p < 0.001 compared with MC group.

The mRNA expression of TLR2, TLR4, MyD88, p65, Occludin1, Claudin1 and MUC-2 in the small intestine was assessed by qRT-PCR to determine whether MSCP modulates the immunomodulatory related genes in mice induced with CTX. The results are shown in **Figure 9**, where CTX treatment caused a significant down-regulation of the seven genes mentioned above (*p* < 0.05). MSCP-L and MSCP-M significantly increased the expression levels of 2 genes (TLR4 and Occludin1) and 4 genes (TLR4, MyD88, p65 and Claudin1), respectively (*p* < 0.05). While MSCP-H and AMP supplements significantly increased the expression of all 7 genes at mRNA level (*p* < 0.05).

### MSCP Attenuated Gut Dysbiosis in CTX-Induced Mice

**Figure 10:**
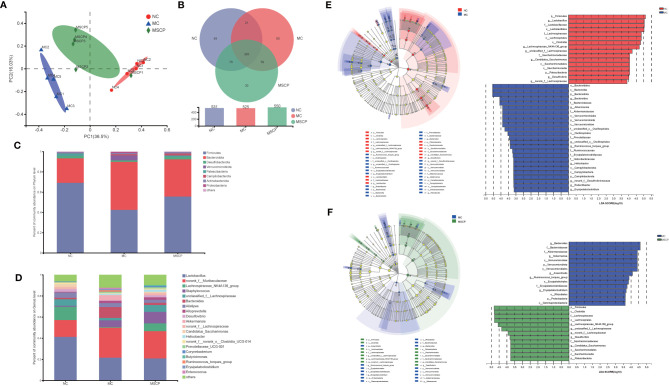
MSCP treatments attenuated gut dysbiosis in CTX-induced mice. **(A)** Principal coordinate analysis (PCoA) of gut microbiota based on bray_curtis distance. **(B)** Similarity and difference of bacteria compositions in different experimental groups at the OTU level. **(C)** Relative abundance of microbiota at the phylum level. **(D)** Relative abundance of microbiota at the genus level. **(E, F)** Difference in dominant microorganisms among each group *via* cladogram and distribution histogram based on LDA. The greater the LDA score was, the more significant the phylotype microbiota was in the comparison. **(E)** NC group and MC group. **(F)** MC group and MSCP group. **(A–F)** Data are pooled in one independent experiment.

To illustrate the regulatory effects of MSCP on gut microflora, we performed high-throughput gene sequencing of 16S-rDNA from fecal bacterial DNA of mice. Since the preliminary results showed a better treatment effect in the MSCP-H group, the NC group, MC group and MSCP-H group (hereafter renamed as MSCP group) were selected for further study. The results of the dilution curves of Shannon diversity index and Chao diversity index indicated that most bacterial diversity and many new phylotypes could be captured (**Supplementary Figures S1A, B**). In order to determine the gut microbiota richness and diversity, we used Ace, Chao1, Shannon, and Simpson indices to evaluate the α-diversity (33) (**Table 3**). By observing Chao1 and Ace indices, both showed that CTX reduced the richness of the microbiota in the cecum of mice. Pleasingly, MSCP can reverse this phenomenon. Shannon indices also showed an increase in both MC and MSCP groups compared to normal mice. The results indicated that MSCP treatment had a moderating effect on the intestinal microbiota and increased bacterial richness and diversity. In order to distinguish the difference in microbial community structure, we calculated the β-diversity of microbial composition using bray_curtis based PCoA (**Figure 10A**). There was a clear separation between the intestinal microbiota of NC and MC mice, while the structure of intestinal flora of mice in MSCP-treated group was closer to that of NC group. The results of the PCoA displayed a noticeable change in the microbial structure after MSCP treatment.

**Table 3:** α-diversity indices of gut microbiota in each group.

Groups	ACE	Chao1	Shannon	Simpson
NC	397.13 ± 42.31	403.26 ± 46.43	3.46 ± 0.16	0.1052 ± 0.031
MC	376.89 ± 32.99	377.66 ± 36.81	3.56 ± 0.24	0.0705 ± 0.023
MSCP	402.00 ± 54.80	411.78 ± 58.24	3.92 ± 0.35	0.0526 ± 0.019

Data are expressed as mean ± SD.

To further investigate the differences in community composition between samples from different treatment groups by using various visualization methods. By plotting the Venn map, the shared richness of gut microflora in each group was analyzed. As shown in **Figure 10B**, 531 OTUs existed in NC group, 525 in MC group, 550 in MSCP group, and 385 OTUs overlap coexisted in the three groups. *Firmicutes* and *Bacteroidota* were the two most dominant phyla at the phylum level, accounting for nearly 90% of the bacterial population, and the next most dominant phylum was *Desulfobacterota* (**Figure 10C**). In this study, the CTX treatment decreased the abundance of *Firmicutes* (*p* < 0.05) and increased *Bacteroidota*, *Verrucomicrobiota* and *Proteobacteria* (*p* < 0.05). To verify the differences in the gut microbiota of some groups, several dominant microbes at the genus level were analyzed and compared. At the genus level (**Figure 10D**), the NC and MSCP groups consisted mainly of *Lactobacillus*, *norank_f_Muribaculaceae Lachnospiraceae_NK4A136_group* and *unclassified_f_Lachnospiraceae*, while the MC group is mainly composed of *Lactobacillus*, *norank_f_Muribaculaceae*, *Staphylococcus* and *Bacteroides*. Compared to NC group, the genera of *Lactobacillus*, *Lachnospiraceae_NK4A136_group* and *unclassified_f_Lachnospiraceae* were decreased and were accompanied by an increase in the genera of *Bacteroides* after CTX treatment (*p* < 0.05). MSCP administration increased the levels of *Lachnospiraceae_NK4A136_group* and *unclassified_f_Lachnospiraceae* and decreased the levels of *Bacteroides* in the CTX-treated mice compared to those in the model group (*p* < 0.05).

In comparing the NC, MC and MSCP groups, LEfSe (LDA > 3.6) analysis was used to determine the key phylotypes of gut microbiota in different groups (**Figures 10E, F**). According to the analysis results, *Firmicutes*, *Lachnospiraceae*, *Lachnospirales*, *Clostridia*, *Lachnospiraceae_NK4A136_group* and *unclassified_f_Lachnospiraceae* displayed high LDA scores, showing that they were abundant OTUs in NC group. In the MC group, *Bacteroidales*, *Bacteroidia*, *Bacteroidota*, *Bacteroides* and *Bacteroidaceae* exhibited higher scores, which indicated that they were significantly affected by CTX. From the composition of MC and MSCP groups, *Firmicutes*, *Clostridia*, *Lachnospiraceae*, *Lachnospirales*, *Lachnospiraceae_NK4A136_group* and *unclassified_f_Lachnospiraceae* in MSCP group recovered to higher levels, similar to NC group. These findings were consistent with the above results. In general, these results indicated that MSCP intervention could ameliorate the imbalance of gut microbiota in CTX-induced immuno-compromised mice to a certain extent, and promoted the proliferation of specific bacteria.

### Effects of MSCP on SCFA Production

**Figure 11:**
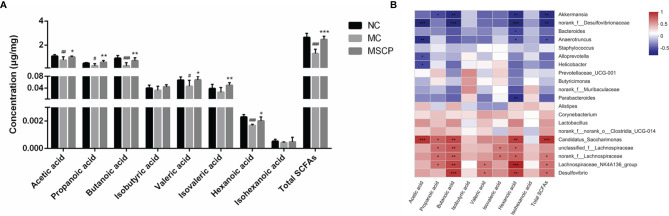
Determination of short-chain fatty acids (SCFAs) in cecal contents. **(A)** The exact amount of SCFAs in cecal contents, including acetic, propanoic, butanoic, isobutyric, valeric, isovaleric, hexanoic and isohexanoic acids, as detected by GC–MS method. **(B)** Heatmap showing Spearman’ correlation coefficient with p < 0.05 between several significant changed fecal SCFA and genera bacteria. Red cells represent positive correlation and blue cells represent negative correlation. Data are expressed as mean ± SD. ^#^*p*< 0.05, ^##^*p* < 0.01, ^###^*p* < 0.001 compared with NC group; ^*^p < 0.05, ^**^p < 0.01, ^***^p < 0.001 compared with MC group.

SCFAs are critical bacterial metabolites in the intestine and mainly consist of acetic acid, propanoic acid, butanoic acid, isobutyric acid, valeric acid, isovaleric acid, hexanoic acid and isohexanoic acid. To evaluate the effect of MSCP on the production of SCFA by microbiota, we determined the SCFAs concentration in the cecal contents by targeted metabolomics assay. From **Figure 11A**, we noticed that the SCFAs spectra for the MC and MSCP treatment groups were different. Specifically, the levels of acetic acid, propanoic acid, butanoic acid, valeric acid, hexanoic acid, and total SCFAs in MC group were significantly lower than those in the control group (*p* < 0.05). Compared with MC group, MSCP could significantly increase the contents of acetic acid, propanoic acid, butanoic acid, valeric acid, isovaleric acid, hexanoic acid and total SCFAs (*p* < 0.05). However, isobutyric acid and isohexanoic acid were not significantly different in content.

Spearman correlation analysis was performed to determine the potential relationship between the major microbiota at the genus level and SCFAs. **Figure 11B** shows that the relative abundance of these bacteria was closely correlated with the metabolites (acetic acid, propanoic acid, butanoic acid, isobuutyric acid, valeric acid, isovaleric acid, hexanoic acid isohexanoic acid and total SCFAs in the cecum content). Thereinto, *Candidatus_Saccharimonas*, *unclassfied_f_Lachnospiraceae*, *norank_f_Lachnospiraceae*, *Lachnospiraceae_NK4A136_group* and *Desulfovibrio* were significantly positively related with SCFAs production, while *Akkermansia*, *norank_f_Desulfovibrionaceae*, *Bacteroides* and *Anaerotruncus* were negatively correlated with SCFAs production. Collectively, MSCP treatment modulated gut microbiota structure and composition, which were associated with metabolite SCFAs level changes.

## Discussion

While cyclophosphamide kills tumor cells, it also kills some rapidly proliferating normal cells (e.g., hepatocytes, intestinal muscular cells) (3, 34, 35). Therefore, host protection from chemotherapy-induced immune damage and dysbiosis is an issue that deserves attention. Currently, we are interested in MSCP due to its superior immunomodulatory properties (26). However, the mechanism by which MSCP ameliorates CTX-induced immunosuppression and intestinal injury *in vivo* has not been reported. In this study, a mouse immunosuppression model using CTX was established to investigate the modulation of immune function and the protective effects of MSCP on intestinal mucosa in mice.

The immune system is composed of immune organs, immune cells, and Immunoreactive molecules, etc. Among these, the thymus and spleen are essential immune organs for lymphocyte differentiation, maturation, and immune response (28, 36). Previous studies have shown that CTX can reduce thymus and spleen indices in mice, and natural polysaccharides can increase these indices, suggesting that polysaccharides can protect the body’s immune organs (37–39). Our results are consistent with previous studies, suggesting that MSCP increases host immune function by stimulating immune organ development.

The intestinal epithelium acts as a physical barrier and plays a vital role in communicating with the microbiota and immune cells (40). The basic functional unit of the intestinal mucosa is the villus-crypt axis, and the villus height/crypt depth (V/C) is also considered to be an essential indicator of intestinal morphology (41, 42). Both *Cordyceps sinensis* polysaccharides and *Sargassum fusiforme* polysaccharides could improve the villi length and crypts depth in CTX-induced intestinal injury mice (22, 43). Consistent with previous findings, CTX-treated mice suffered severe damage to intestinal villi and crypts structures, which was alleviated by MSCP intervention in a dose-dependent manner. Goblet cells are specialized secretory IECs that secrete various mucins that form a mucus layer on the surface of the epithelium to defend against microbial invasion (12, 44, 45). AB-PAS staining showed that MSCP increased the number of goblet cells and mucin area in the jejunum. This was similar to the amelioration of goblet cells and mucin by synergistic American ginseng polysaccharides and ginsenosides (40). Collectively, MSCP improved the irregular morphology of intestinal tissues by attenuating CTX-induced intestinal stress injury.

The cytokines are a series of soluble proteins produced by immune cells. They play an important role in immune cell differentiation and tissue regeneration (46). Earlier studies have shown that some polysaccharides can activate immune cells and upregulate cytokine levels to reduce CTX-induced immune damage. *Auricularia auricula* polysaccharides ameliorate CTX-induced immunosuppression in mice by increasing serum IFN-γ、IL-2、IL-4、IL-10 and TNF-α production (29). *Cordyceps sinensis* polysaccharides produced IL-17, IL-21, and TGF-β3 in the small intestine, which modulated the immune response to CTX induced intestinal mucosal injury in mice (47). The present study found that MSCP could promote cytokine secretion to regulate systemic immune function and alleviate intestinal mucosal injury to some extent. The intestine is the body’s largest immune organ, and the intestinal immune system can produce cytokines that migrate to the peripheral lymph nodes and spleen, which can trigger a systemic immune response (48, 49). Therefore, we speculated that MSCP showed a systemic immunostimulatory effect on immunosuppressed mice by triggering intestinal mucosal immune responses.

Toll-like receptors (TLRs) are pattern recognition receptors that exist in intestinal immune cells (50). TLRs and glycosyl ligands bind and signal to MyD88, which further activates the NF-B signaling pathway and induces the release of various cytokines, ultimately triggering an immune response (51). p65 regulates the transcription and secretion of cytokines in the NF-κB signaling pathway, and its expression is closely related to immunity (20). We observed that MSCP restored the expression of TLR2, TLR4, MyD88, and p65 in MC group mice to some extent, especially in the MSCP-H group. It is suggested that the high dose of MSCP (400 mg/kg/d) is more favorable to activate NF-κB pathway. Intestinal epithelial barrier function is also regulated by tight junction proteins (TJ), and Occludin1 and Claudin1 are crucial tight junction proteins whose normal expression is vital for maintaining the integrity of intestinal epithelial tight junctions (52). MUC-2 is an intestinal type mucin secreted by goblet cells to form intestinal mucus that resists bacterial destruction (53). In addition, it has been reported that epithelial MyD88 signaling targeting deficiency leads to an increase in the number of mucus-associated bacteria and downregulation of MUC-2 expression (54). In this study, we found that MSCP upregulated the expression of Occludin1, Claudin1, and MUC-2 in the intestinal mucosa, thereby attenuating CTX-induced intestinal barrier damage in mice.

A wide variety of microorganisms are present in the gastrointestinal tract of mammals. The intestinal microbiota is vital for host growth, immune function, resistance to infection, and metabolism (48, 55). Multiple studies have demonstrated the vital role that polysaccharides play in regulating gut microbiota to modulate host immunity (21). Our results demonstrated that MSCP increases the α diversity index of microbial communities. Various studies have found that low microbiota diversity and species levels are associated with abnormal immunity (56). According to PCoA analysis, CTX treatment altered the overall microbiota structure, and MSCP could mitigate this negative effect, with the structure of the mouse flora in the MSCP group more similar to that of the NC group.

Immediately after, a detailed comparative analysis of the gut microbiota at different taxonomic levels (phylum and genus). *Firmicutes* and *Bacteroidota* are the two most common phyla that can utilize polysaccharides by generating carbohydrate-active enzymes (57). Interestingly, in our study, the MC group showed a significant decrease in the relative abundance of *Firmicutes* and a significant increase in the relative abundance of *Bacteroidota* compared to the NC group. This suggests that *Bacteroidota* may also use CTX to produce immunosuppressive effects in mice, consistent with the previously reported regulatory effects of *Caulerpa lentillifera* polysaccharides and polysaccharides from bee collected pollen of Chinese wolfberry (29, 58). At the genus level, *Lactobacillus* is one of the common probiotics that affect the interaction of CD^+^ T cells with dendritic cells, lymphocyte proliferation and cytokine secretion to exert mucosal immune responses (59). *Lactobacillus* decreased significantly after CTX treatment in mice, indicating that CTX has the potential to inhibit its proliferation. However, there was no difference in the relative abundance of *Lactobacillus* in the MSCP group compared with the MC group, probably due to the low utilization of MSCP by *Lactobacillus*. The abundance of both *Lachnospiraceae_NK4A136_group* and *unclassified_f_Lachnospiraceae* was significantly lower in the MC group than in the NC group. However, the administration of MSCP resulted in an improvement. *Lachnospiraceae* is a variety of SCFA-producing bacteria such as acetic, propionic and butyric acids that inhibit toxin production by *Clostridium difficile* (11, 60). *Lachnospiraceae* has been reported to play a significant role in the degradation of polysaccharides, therefore, degrade MSCP (61). In addition, MSCP administration increased the levels of acetic acid, propionic acid, butyric acid, valeric acid, isovaleric acid, and capric acid. This is similar to previous studies that *Wild morels* polysaccharides and Lotus Seed Resistant Starch both augmented SCFAs levels in mice by increasing the content of SCFAs-producing *Lachnospiraceae* (56, 62). Many polysaccharides have been confirmed to cause increased expression of *Bacteroides* (63–66). Nevertheless, different sources of polysaccharides have different effects on the gut microbiota. In the present study, *Bacteroides* levels were increased in the MC group compared with the NC group, and it is speculated that CTX may also be heavily utilized by this bacterium, while the relative abundance of *Bacteroides* was similar in the MSCP group to that in the NC group. The LEfSe analysis showed that the central microbiota of the MSCP group were *Firmicutes*, *Clostridia*, *Lachnospiraceae*, *Lachnospirales*, *Lachnospiraceae_NK4A136_group* and *unclassified_f_Lachnospiraceae*, in keeping with the critical phylotypes of the gut microbiota of mice in the NC group. It is suggested that MSCP treatment can modulate the bacterial phylotypes belonging to *Firmicutes*. As producers of acetic, propionic and butyric acids in our intestinal tract, Firmicutes are the primary degraders of indigestible polysaccharides (67). The study showed that polysaccharides from *Artocarpus heterophyllus Lam*. pulp modulate intestinal flora in mice by increasing Firmicutes abundance (68). It has been reported that immune compromise is necessarily closely related to metabolic disorders, especially reducing the levels of SCFAs, which have mucosal protective effects (69). We previously noted that the MSCP group increased the content of SCFAs in the intestine of mice. Correlation analysis showed that *Candidatus_Saccharimonas*, *Uncassed_f_Lachnospiraceae*, *norank_f_Lachnospiraceae*, *Lachnospiraceae_NK4A136_Group* and *Desulfovibrio* were positively correlated with the production of SCFAs. In summary, the results of this study confirmed that MSCP as prebiotic improved microbial community diversity and regulated the relative abundance of dominant microbiota from the phylum level to the genus level and that these bacteria could depolymerize and metabolize polysaccharides to produce SCFAs, resulting in beneficial effects on the organism.

## Conclusion

The aim of this experiment was to investigate the protective effects of MSCP on CTX-induced intestinal barrier damage and immunosuppression in mice. The results confirmed that MSCP was effective in improving body weight, protecting immune organs and enhancing the production of immune-related cytokines. Enhancement of intestinal health was the key way of MSCP to modulate the host’s immunoreactions. MSCP effectively recovered impaired mucosal integrity, regulated the expression of intestinal immune-related genes, and activated the intestinal TLRs/MyD88/NF-κB p65 pathways. Moreover, MSCP reversed the level of SCFAs and restored CTX-induced gut microbial dysbiosis by improving microbial community diversity and modulating microbial community structure and composition. On the basis of these findings, a theoretical foundation was provided for the development of functional foods based on *M. speciosa* as well as agents to protect against side effects caused by cancer chemotherapy.

## Data Availability Statement

The datasets presented in this study can be found in online repositories. The names of the repository/repositories and accession number(s) can be found below: https://www.ncbi.nlm.nih.gov/, PRJNA759356.

## Ethics Statement

The animal study was reviewed and approved by The Institutional Animal Care and Use Committee of Guangxi University (Nanning, China).

## Author Contributions

XC, WS, JL, and HS conceived the study and designed the project. XC, BX, EW, YC, KH, GZ, CZ, and YX performed the experiment. XC, WS, BX, and EW analyzed the data. XC and WS drafted the manuscript. JL and HS revised the manuscript and supervised the whole study. All authors contributed to the article and approved the submitted version.

## Funding

The Key Research and Development Plan of Guangxi, China (AB19245037), Natural National Science Foundation of China (317607446) and the Major R&D Project of Nanning Qingxiu District (2020005).

## Conflict of Interest

The authors declare that the research was conducted in the absence of any commercial or financial relationships that could be construed as a potential conflict of interest.

## Publisher’s Note

All claims expressed in this article are solely those of the authors and do not necessarily represent those of their affiliated organizations, or those of the publisher, the editors and the reviewers. Any product that may be evaluated in this article, or claim that may be made by its manufacturer, is not guaranteed or endorsed by the publisher.
