# Supplementary material for: Polysaccharides From the Roots of Millettia Speciosa Champ Modulate Gut Health and Ameliorate Cyclophosphamide-Induced Intestinal Injury and Immunosuppression
Source: Front Immunol. 2021 Oct 21;12:766296. doi: 10.3389/fimmu.2021.766296 (PMC8567740; doi:10.3389/fimmu.2021.766296)
Supplement: Supplementary file 1 [file DataSheet_1.pdf]

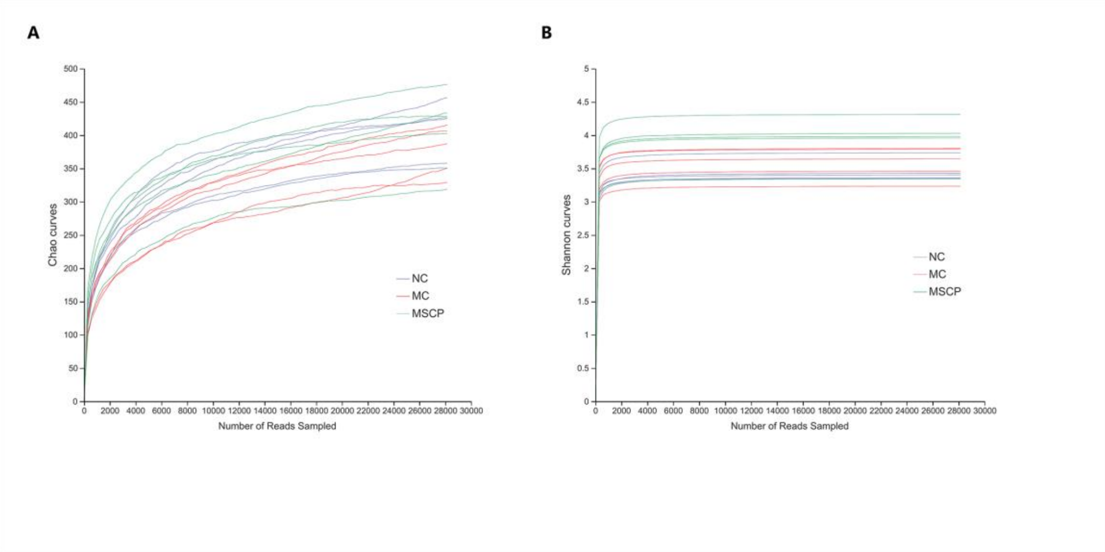

Supplementary Figure 1. Alpha diversity indexes of microbiota in different groups. **(A)** Chao diversity index. **(B)** Shannon diversity index.
