# Supplementary material for: Polysaccharides From the Roots of Millettia Speciosa Champ Modulate Gut Health and Ameliorate Cyclophosphamide-Induced Intestinal Injury and Immunosuppression
Source: Front Immunol. 2021 Oct 21;12:766296. doi: 10.3389/fimmu.2021.766296 (PMC8567740; doi:10.3389/fimmu.2021.766296)
Supplement: Supplementary file 2 [file Table_1.pdf]

Supplementary Table S1. Primer sequences of mRNA for qRT-PCR.

Genes	Primer sequence (5'-3')
β-actin	F: GTGCTATGTTGCTCTAGACTTCG
	R: ATGCCACAGGATTCCATACC
TLR2	F: ACCCGCCCTTTAAGCTGTGT
	R: TCGTACTTGCACCACTCGCT
TLR4	F: TCTGGGGAGGCACATCTTCT
	R: AGGTCCAAGTTGCCGTTTCT
MyD88	F: TCATGTTCTCCATACCCTTGGT
	R: AAACTGCGAGTGGGGTCAG
p65	F: GCCTCTGGCGAATGGCTTTA
	R: TGCTTCGGCTGTTCGATGAT
Occludin1	F: GCCCCTCTTTCCTTAGGCG
	R: AAGATAAGCGAACCTGCCGA
Claudin1	F: TCTACGAGGGACTGTGGATG
	R: TCAGATTCAGCTAGGAGTCG
MUC-2	F: CCCAGAAGGGACTGTGTATG
	R: TGCAGACACACTGCTCACA
